# The experiences of merging health insurance funds in South Korea, Turkey, Thailand, and Indonesia: a cross-country comparative study

**DOI:** 10.1186/s12939-021-01382-w

**Published:** 2021-02-26

**Authors:** Mohammad Bazyar, Vahid Yazdi-Feyzabadi, Arash Rashidian, Anahita Behzadi

**Affiliations:** 1 Department of Health Promotion, Faculty of health, Ilam University of Medical Sciences, Ilam, Iran; 2 National Center for Health Insurance Research, Iran Health Insurance Organization, Tehran, Iran; 3 Social Determinants of Health Research Center, Institute for Futures Studies in Health, Kerman University of Medical Sciences, Kerman, Iran; 4 Evidence and Research Department, World Health Organization Regional Office for the Eastern Mediterranean, Cairo, Egypt; 5 Health Services Management Research Center, Institute for Futures Studies in Health, Kerman University of Medical Sciences, Kerman, Iran

**Keywords:** Health insurance, Fragmentation, Merging, Equity, Risk pooling, Single insurance

## Abstract

**Background:**

Fragmentation in health insurance system may lead to inequity in financial access to and utilization of health care services. One possible option to overcome this challenge is merging the existing health insurance funds together. This article aims to review and compare the experience of South Korea, Turkey, Thailand and Indonesia regarding merging their health insurance funds.

**Methods:**

This was a cross-country comparative study. The countries of the study were selected purposefully based on the availability of data to review their experience regarding merging health insurance funds. To find the most relevant documents about the subject, different sources of information including books, scientific papers, dissertations, reports, and policy documents were studied. Research databases including PubMed, Scopus, Google Scholar, Science Direct and ProQuest were used to find relevant articles. Documents released by international organizations such as WHO and World Bank were analyzed as well. The content of documents was analyzed using a data-driven conventional content analysis approach and all details regarding the subject were extracted. The extracted information was reviewed by all authors several times and nine themes emerged.

**Results:**

The findings show that improving equity in health financing and access to health care services among different groups of population was one of the main triggers to merge health insurance funds. Resistance by groups enjoying better benefit package and concerns of workers and employers about increasing the contribution rates were among challenges ahead of merging health insurance funds. Improving equity in the health care financing; reducing inequity in access to and utilization of health care services; boosting risk pooling; reducing administrative costs; higher chance to control total health care expenditures; and enhancing strategic purchasing were the main advantages of merging health insurance funds. The experience of these countries also emphasizes that political commitment and experiencing a reliable economic growth to enhance benefit package and support the single national insurance scheme financially after merging are required to facilitate implementation of merging health insurance funds.

**Conclusions:**

Other contributing health reforms should be implemented simultaneously or sequentially in both supply side and demand side of the health system if merging is going to pave the way reaching universal health coverage.

## Introduction

In many countries with health insurance system, various insurance schemes coexist which lead to the fragmentation of health insurance schemes and reduction of risk pooling [1]. This fragmentation creates concerns regarding equity in access to health services among different groups of population as well as financial instability for small health insurance funds (HIFs) [2]. Fragmentation can also postpone reaching universal health coverage’ objectives such as reduction the financial burden as it reduces the potential degree of risk redistribution from a given amount of prepaid funds [3]. Fragmentation as a concern in health insurance system [4, 5] has been addressed in World Health Organization’ (WHO) reports 2000, 2008 and 2010 [6–8]. Also, the World Bank has warned the Eastern Asian countries against the high administrative costs of multiple health insurance funds, duplication of benefit packages, and loss of negotiation power with health services providers [2].

Because of that, one of the main concerns in health financing reforms which most countries are looking for is a guide for proper implementation of single and multi-payer health insurance systems. In the single-payer system, one organization, which is usually the government, carries out the tasks of collecting and pooling revenues, and purchasing health services for the entire population of the country while in multi-payer systems these tasks are done by several organizations. In the single-payer system, there is a single health insurance fund and all financial risks are pooled within it while in multi-payer systems there are multiple health insurance funds which lead to fragmentation in risk pools. The single-payer system has the exclusive power for purchasing services, but in multi-payer systems, people belong to different health insurance organizations [9, 10].

Studies show that achieving universal health coverage for the entire population requires various forms of cross-subsidies including the subsidy shift from the rich to the poor, from healthy to unhealthy people, and from low-risk persons like the young to the high-risk ones such as adults and the elderly. Many middle-income countries such as Brazil, Indonesia, Philippines, Turkey, and South Korea are trying to reduce the disparity in accessing health service through providing universal health coverage, enhancing health insurance funds based on pre-payment procedures and implementing health system enhancement programs [11].

In countries in which there are official and compulsory HIFs as well as HIFs with a significant number of beneficiaries, integrating and merging the existing HIFs is one possible option to boost health insurance system and move towards achieving universal health coverage [12]. This policy is more applicable in countries where there is no competition among health insurance schemes or moving towards competition is expensive as the required conditions are not available [13]. Some countries have applied this approach for extending the size of HIFs, improving equity and justice, and improving risk pooling [12, 14].

In other countries, health reforms have led to a decrease in the number of HIFs. For instance, in Estonia, in 2001 and in Poland, in 2003, 17 regional HIFs were merged to form a single national fund [15].

Indonesia and Peru are also moving toward integration of risk pools as the last step in universal health coverage. In November 2011, Indonesia passed a law which obligated the five governmental HIFs which covered separate population groups, to merge under a comprehensive program aimed to benefit from cross subsidy, reducing administrative costs and providing more equitable benefit packages [16]. Indonesia officially implemented the National Health Insurance program on January 1, 2014. This program consists of the merger of programs which were previously covered by government and other social security insurance programs, but now will become a single-payer and cover all citizens [14]. In Peru, the Universal Health Insurance Law of 2010, created an obligatory framework for achieving universal health coverage through coordinated process of merging two main HIFs [14]. Also, Turkey undertook extensive reforms in order to merge different insurance schemes and achieved integration and cross-subsidization. Brazil merged different programs under Unified Health System according to constitution of 1988 which was financed through general taxation. Thailand merged two main programs under the universal health coverage in 2001 and with the highest number of insured members being in this fund, cross-subsidization and financial security were guaranteed. However, there are still three HIFs in Thailand and the per capita cost across these HIFs is very unstable due to the lack of redistribution of resources. Ghana has taken some measures towards HIFs integration. Ghana has a single risk pool under the national health insurance, which transfers resources from the rich to the poor through progressive general taxation and the redistributive function of the national health insurance program. In Vietnam, efforts to integrate HIFs are still ongoing. However, the real pooling of resources and distribution of costs are not fully implemented [14].

Although success of health reforms is dependent on how well they are developed and implemented technically, they are facilitated or inhibited by a wide range of social, economic, political and cultural factors which is different from one country to another. This clarifies that moving toward to single-payer in health insurance is context-sensitive and cross-country comparison among different countries provide proper evidence for policy makers across the world particularly developing countries.

It’s worth mentioning that in a fragmented health insurance system, the degree of differences among HIFs in following items can affect directly the state of health equity and hinder implementation of merging HIFs. These items are as follows: the percent of the whole population under coverage of each health insurance scheme; the extent of differences in the contribution rates, benefit package, quality of health care received by members of different risk pools, and more importantly variations in user charges and amount of out-of-pocket expenditures paid by different beneficiaries belong to different insurance schemes for the same health services they use [1].

These differences among risk pools in countries even with only several insurance schemes such as Turkey and Thailand and Indonesia made policy makers to move towards merging HIFs as a solution to end up inequity in health care services. In contrast in some members of Organization for Economic Co-operation and Development (OECD) with multiple risk pools such as the Slovak Republic, the Czech Republic, Switzerland and France the performance of the health insurance system in terms of equity in above indicators is satisfactory as health insurance schemes are mandated to provide the same benefit package by getting the same contribution rates, so no matter which scheme the beneficiaries belong to, they access to the same health services [17].

The experience of countries which have moved toward consolidation of HIFs as a strategy to achieve universal health coverage, especially in recent years, could be very useful for developing countries and countries which are looking for ways of enhancing and extending their health insurance systems. This is due to the fact that identifying operational challenges and obstacles in the process of merging of HIFs and its advantages and disadvantages will provide proper evidence for policy making in this sector as well as useful lessons for moving toward the funds merging policy. Therefore the present study was conducted aiming at a comparative analysis of the experiences of four Asian countries namely, South Korea, Turkey, Thailand and Indonesia in establishing national unified health insurance scheme or reducing the number of HIFs.

## Methods

### Search strategies and selection criteria

The present cross-country comparative study was a part of larger study about policy analysis of merging health insurance funds in Iran conducted in 2014. This review study was conducted aiming to investigate and learn lessons from the experiences of other countries regarding the policy of merging and reducing the number of health insurance funds.

To find countries with experience of merging HIFs and the most relevant documents covering the subject of merging HIFs or reducing fragmentation in health insurance system, different sources of information were searched including books, scientific papers, dissertations, reports, and policy documents. Formal reports and documents published by international organizations such as WHO and World Bank were studied as well. To find scientific articles and dissertations regarding the subject, the following databases including PubMed, Google Scholar, Science Direct and ProQuest were searched using key words merger, combination, consolidation, or amalgamation of health insurance funds, fragmentation in health financing, multiple health insurance, single health insurance, multiple and single payer, and risk pooling. At the first quick review, the title and abstract of extracted documents scanned looking for information directly related to merging health insurance funds. First round was not satisfactory as the literature to address the merging HIFs directly was not rich enough. So in the second round more general key words including health insurance, national health insurance, social health insurance, health insurance reform, health system reform, and health financing used to find relevant documents emphasizing on countries that had planned and implemented national health insurance reforms over the last few decades aiming to merge their health insurance funds or moving towards reduction of fragmentation in health insurance system. All documents extracted in both the first and second round of search were scanned more deeply searching for countries with health insurance reforms over the last decades. A list of 13 countries namely Indonesia, Estonia, Poland, Lithuania, Czech Republic, South Korea, Colombia, Turkey, Ghana, Peru, Brazil, Greece, and Thailand were found in the literature that had implemented merging health insurance funds, currently working on it or planning to merge their HIFs. In the next step, all of those countries were searched individually and all documents regarding health insurance reforms were extracted. Finally we chose four countries of South Korea, Turkey, Thailand and Indonesia purposefully for the review as there was more information in the literature about their experience regarding merging HIFs especially about reasons, process, challenges, and advantages of merging HIFs. We focus exclusively on English language documents. Although few available Persian documents were searched and reviewed which were mainly translation of English documents. No time limitation was applied to find relevant documents and the last update for finding the documents was conducted from 20 to 30 of March, 2020. The bibliographies of the most relevant articles and documents were searched and further documents were found and retrieved. We also communicated with the authors of the most relevant articles personally via email from the selected countries asking them to introduce other probable missing relevant articles or publications regarding the subject. It was helpful in finding more several documents.

### Data analysis

All documents were scanned by one reviewer (MB) and documents with relevant details about the subject studied meticulously. The content of documents was analyzed using a data-driven conventional content analysis approach and all details regarding the subject were extracted [18]. The five-stage process of qualitative data analysis was done: understanding (familiarization), identifying a thematic framework (thematic), coding (indexing), charting and mapping, and interpretation [19]. The extracted information was reviewed in a group by research team and categorized by all authors several times and the main themes were finalized. The extracted information was categorized in 9 themes. It’s worth mentioning that in reviewing the experience of each country regarding the topic, we aimed to act both retrospectively and prospectively. It means that we focused on the real and potential consequences according to the experience of each country. We mentioned whatever occurred during and after merging HIFs and also the potential events that may happen in the future as a result of merging HIFs which need to be considered as well.

Searching and screening the documents was conducted by two members of the research teams (MB & VYF). Frequent sessions were held among these two authors and the retrieved resources were checked by four criteria proposed by Jupp (1996) including authenticity (being original and genuine), credibility (accuracy), representativeness (being representative of the totality of the documents in their class) and meaning (what they say). It should be noted, as this study employed a narrative approach, all related documents for four countries irrespective type of study, were included for the analysis.

The trustworthiness of results was enhanced by using four criteria proposed by Lincon & Guba including credibility, transferability, dependability, and confirmability.

To ensure credibility, triangulation of sources technique was employed. As different data sources including grey literature, published articles retrieved from scientific and valid databases, international and national organizations’ websites were deeply approached. This helped to the examination, conformation, supplementation, and consistency of different data sources. Moreover, the primary findings extracted from the sources were reviewed by the research team with a relatively rich background in health insurance field. We also tried to incorporate all elements of the data even those that do not support or appear to be different with the dominant pattern of the data.

In case of transferability, different context of four countries were described as much as necessary for contribution to the knowledge of merging funds of health insurances. Furthermore, the details of selection countries, data gathering, screening, and data analysis were thickly described.

To ensure the dependability, one of authors (AB) acted as an external auditor and reviewed the findings in order to evaluate whether or not the findings, interpretations and conclusions are supported by the data.

In case of confirmability, all perspectives related to agreement or disagreement with merging the health insurance funds were welcomed and tried to keep away bias arisen form one specific belief about research [20, 21].

## Results

The findings related to the experience of merging health insurance funds were categorized in the following 9 themes: the structure of health insurance system before and after the merging; the reasons behind moving towards merging health insurance funds; the process of merger; the facilitators of merging; the resistance against and limitations ahead of the merger; the positive consequences of merger; the negative consequences of merger; the simultaneous and contributing health reforms alongside merger; and finally the future challenges. The above 9 themes were used to narrate the experience of each country separately for easier understanding. The findings related to merging health insurance funds for the South Korea, Turkey, Thailand and Indonesia are presented respectively. Table 1 shows the main health financing indicators for the studies countries.

### South Korea

#### Health insurance system in South Korea before and after merging

Before the merger of all insurance schemes in Korea in 2000, there were three social health insurances for: (1) government employees and teachers and their dependents (one health insurance fund), (2) industrial workers and their dependents with nearly 140 insurance schemes and (3) the self-employed and workers in firms with less than 5 workers, with nearly 230 insurance schemes (92 HIFs in rural and 135 HIFs in urban areas). Also, there was another scheme called Medicaid for the poor which covered 3–5% of the remaining population [22]. Health insurance for employees was based on where they worked and, for the self-employed, on where they lived. For the health insurance scheme of employees, large companies had their own insurance while small and medium-sized companies were a part of insurance schemes existing in their geographical area [22]. Table 2 shows more details of the health insurance system.

**Table 1:** General characteristics of health financing indicators in the studied countries

	Turkey	South Korea (2011)	Thailand (2012)	Indonesia (2014)
Classification by income level	Upper-middle-income	High-income	Upper-middle-income	-Lower-middle-income
Gross national income per capita (PPP international $, 2013)	**18,760**	**33,440**	**13,510**	**9260**
Total expenditure on health per capita (Intl $, 2014)	**1036**	**2531**	**600**	**299**
Total health expenditure as % of GDP	6.1	7.4	4.5	2.8
Public expenditure on health as % of total expenditure in health	73	55.3	76	37.8
Private expenditure on health as % of total expenditure in health	27–37	44.7	24	62.2
Government health spending as % of total government spending	9.8–13.9	15.2	11	5.7
Out-of-pocket (OOP) payments as % of total expenditure on health	17.4	35.2	11.6	46.9

**Table 2:** Social health insurance structure in South Korea before and after the merger

		Scheme	Population eligibility	Benefit package	Financing	Affiliation
**South Korea**	**Before**	(A single) social health insurance scheme, 1979	government employees and teachers and their dependents (based on employment)	The benefit package of health insurance mainly includes curative services, but includes biannual health checkups and vaccination is provided free of charge in public health centres Benefit package was the same for all in South Korea even before the merger of insurance societies in 2000	For industrial workers and government and school employees, contribution is proportional to wage income and shared equally between the employee and employer. Before the merger of insurance societies in 2000, the average contribution rate was 5.6% (of wage income) for government and school employees.	the Ministry of Health and Welfare
		140 social health insurance schemes, 1977	for industrial workers and their dependents	″	For industrial workers and government and school employees, contribution is proportional to wage income and shared equally between the employee and employer. 3.75% for industrial workers, with a range of 3.0–4.2% depending on the insurance society	the Ministry of Health and Welfare
		about 230 health insurance societies (92 in rural and 135 in urban areas), 1981	for the self-employed and workers in firms with less than five employees, based on residential area	″	-	the Ministry of Health and Welfare
		Medicaid, 1977	for the poor (3–5% of the population)	″	-	the Ministry of Health and Welfare
	**After**	the National Health Insurance Corporation (NHIC), 2000	All population	″	As of 2006, the contribution rate was 4.48% (NHIC 2007).

**Table 3:** Social health insurance structure in Turkey before and after the merger

		Scheme	Population eligibility	Benefit package	Financing	Affiliation
**Turkey**	**Before**	SSK (the Social Insurance Organisation), 1945	blue collar workers (49.49%)	Pre-paid short-term medical and maternal benefits, employment related accident and occupational disease benefits; long-term benefits for old age, disability and survivor pensions; did not provide or pay for preventive services	Employees (5% of salary), employers (6%), state subsidized (8.5% employer share 5% employee share)	Attached to the Ministry of Labour and Social Security until May 2006, transferred to the Social Security Institution
		Bağ-Kur, 1971	self-employed people, artisans, and organised groups (23.43%)	All outpatient and inpatient diagnosis and treatment. The insured were required to pay health insurance premiums for at least 8 months and have no record of default of health insurance and long term insurance premiums	20% premiums collected from beneficiaries. The scheme worked on a reimbursement system	Attached to the Ministry of Labour and Social Security until May 2006, transferred to the Social Security Institution
		Emekli Sandigi (the Government Employees Retirement Fund), 1949	Retired civil servants and their dependents (13%)	Diagnosis and treatment	20% of the deduction of the Government Employees Retirement Fund (State share as employer), 16% of the deduction of the Government Employees Retirement Fund (participant share) for both health and pension; funded through the contributions of the active civil servants and their employers (general budget revenues)	Attached to the Ministry of Labour and Social Security until May 2006, transferred to the Social Security Institution
		the Active Civil Servants Insurance Fund, 1965	civil servants in work and their dependents	Diagnosis and treatment	Benefits were financed by general tax revenues; no premiums were assessed for active civil servants while they were covered directly through their employers	Attached to the Ministry of Finance through their institutions until 2010, transferred to the Social Security Institution in January 2010
		the Green Card, 1992	Uninsured poor individuals (15%)	Inpatient and ambulatory care, pharmaceuticals	General budget (100%)	Attached to the Ministry of Finance through the Ministry of Health, will be transferred to the Social Security Institution by the end of 2012
	**After merging**	General Health Insurance scheme, 2006	Turkish citizens, refugees, foreigners residing in Turkey for more than 1 year	(1) Primary care, rehabilitation, preventive services; (2) ambulatory and inpatient care; (3) maternal benefits as well as in vitro fertilization treatment; (4) partial general oral and dental care; (5) blood and blood products, bone marrow, vaccination, medicine, medical devices and equipment	12.5% of a person’s gross income, and employee (5%), and employer (7.5%) salary deductions. The rate for people who are only dependent on General Health Insurance Scheme is 12% of their earnings. The contribution of the state will be 3% of insured earnings as the basis for premiums	Attached to the Ministry of Labour and Social Security through the Social Security Institution

**Table 4:** Social health insurance structure in Thailand before and after the merger

		Scheme	Population eligibility	Benefit package	Financing	Affiliation
**Thailand**	**Before**	Medical Welfare Scheme, 1975	low-income individuals, elderly (those older than 60) and all children younger than 12, the disabled, monks and religious leaders, and war veterans (32% in 1999)	free medical care at public facilities: outpatient care, inpatient care, diagnostics, and medicines, Annual physical checkup (41% of the whole population in 1997)	general tax revenue	Ministry of Public Health
		Civil Servant Medical Benefit Scheme (CSMBS), 1980	Civil servants themselves, their parents, spouses and up to three children under 18 years old (9%)	Inpatient services, outpatient services, Annual physical checkup	general tax revenue	Ministry of Finance
		the Social Security Scheme (SSS), 1990	Formal workers in firms with over 10 employees for non-work related sickness, maternity, invalidity and funeral grants (7%)	Inpatient services, outpatient services, prevention, health promotion	compulsory tripartite (employers, employees and the government) contributions of 1.5% of payroll each	Ministry of Labor
		The Health Card Project (HCP), 1983	Covers non-poor households, mostly in rural areas, who can voluntarily buy a card which attracts a matching tax subsidy and which gives them access to free care at public facilities as long as they follow referral channels (16% in 1999)	Inpatient services, outpatient services, Annual physical checkup, prevention, health promotion	Household: B 500 + per year, government subsidy B 1000 per year	Ministry of Public Health
	**After**	The Universal Coverage scheme (2002)	All the uninsured, voluntary health card holders, and those covered by the medical welfare scheme under a single unified program (47 million, 74% whole population)	Inpatient services, outpatient services, prevention, health promotion	General tax, non-contributory	National Health Security Office (NHSO)
		CSMBS	As before	As before	As before	As before
		SSS	As before	As before	As before	As before

**Table 5:** Social health insurance structure in Indonesia before and after the merger

		Scheme	Population eligibility	Benefit package	Financing	Affiliation
**Indonesia**	**Before**	Civil Servant Social Health Insurance Scheme (Askes), 1968	All civil servants and pensioners of civil servants and military personnel	All members entitle to comprehensive benefits considered medically necessary	2% of their basic monthly salary, regardless of their marital or family status	State own company
		Jamsostek, 1992	Social Health Insurance Scheme for Private Employee	The benefits are in kind, provided through various health care providers contracted. Other Jamsostek programs pay cash benefits to the beneficiaries.	Only employers are mandated to pay premium of 3% (singles) and 6% (married) of employees	–
		Commercial Health Insurance	They are actually non insurance companies selling health insurance to	comprehensive health services	–	Run by the owner
		*Jamkesmas* (by national government),2008	Poor people	Providing different block grants of financial assistance for poor family to access health care services	Public budget	-
		*Jamkesda* (by regional government), 2008	Poor people	Providing different block grants of financial assistance for poor family to access health care services	Public budget	-
	**After**	national health insurance programme (BPJS), 2014	All citizens	comprehensive basic benefit package, outpatient and inpatient care at primary level up to tertiary hospital level	5% of salary of salaried workers and their family members (Employers, with contribution from employees); 5% of monthly pension for pensioners, 5% of 45% of civil servant basic salary for Veterans or their widows; Public budget for poor people,	National Social Security Agency (Badan Penyelenggara Jaminan Sosial – BPJS), BPJS Health for health-care [51]

#### Policy objectives: reasons behind moving toward merging of HIFs in South Korea

In South Korea, health financing was not reformed to control or reduce costs; but the inequity in financing and differences in financial capacities of HIFs and other problems discussed in following were among the main concerns for undertaking reforms [13]. Before merging, the social health insurance organizations reimbursed the providers based on a fixed fee schedule. This forced the providers to resort to cost-shifting and imposed higher fees on the uninsured persons which in turn led to exacerbating the inequity in access to health services [22]. Inequity in health financing across different income and job groups, differences in financial capacity across HIFs, and chronic financial instability of some rural HIFs stimulated the foundational change in the national insurance structure [13]. Before the merger, many of HIFs were small and unable to integrate their resources effectively. Also, due to the lack of competition, these HIFs did not have any tendency to merge and improve risk pooling [13, 23]. Moreover, for the self-insured members in poor areas, the portion of income assigned to insurance, was greater than it was in rich areas. There was horizontal inequality since in some areas people with a similar income would pay different contributions for the same benefits based on their insurance type [13]. The government introduced health insurance for the self-employed due to the inequity in payments for health care services between the insured and the uninsured. In 1998, before the merger, health insurance schemes for the self-employed gained 10.9% of their total revenue from risk equalization fund, however financial problems of HIFs in some rural areas were still an issue. Therefore, the government decided to merge all of the schemes in 2000 [22].

#### Process of the merger

In South Korea the process of merging began with HIFs for self-employed workers, teachers and government employees in October 1998 and by merging these schemes together the National Health Insurance Corporation (NHIC) was created. In July 2000, the HIFs for industrial workers were merged with the NHIC, and the national health insurance of Korea became a single payer insurance system. The NHIC had separate HIFs for government and school employees, industrial workers and the self-employed. The HIFs for industrial workers and government and school employees were merged together in 2001 and this new fund was merged with the fund of the self-employed in 2003. Therefore, the single payer system was established in 2003 [13]. In Korea, ass claim review and payment to health care providers were centralized even before the merger and all health insurance schemes followed the same statutory benefit packages, the process of merging didn’t face challenges in these areas [22].

#### Facilitators of the merger

##### Leadership and political commitment

In South Korea, several factors were influential in facilitating the process of merging. The main political contributing factors were the president, Chun Doowhan, and the presidential candidate of the ruling party, Roh Taewoo, who were looking for political support and legitimacy, suggested the universal health insurance coverage [23]. The political regime and motivation for political legitimacy played key role in the introduction and development of the social health insurance. Politicians who had legitimacy in rural areas advocated the merger of insurance schemes. Farmers, the poor in urban areas, academics and civil groups were also among the early advocates of uniform financing systems [22]. The residence-based municipal health insurance societies that were suffering from serious financial problems due to covering lower-income and older people and trade unions in the mid-1990s that focused on the redistribution and solidarity across different income groups rather than occupational differences were among the supporters of merging health insurance schemes [24].

##### Neutrality of potential opposing groups such as physicians

In South Korea, the neutrality of these groups helped the structural changes in health insurance system [13]. Physicians were neutral as they were not directly affected by the outcomes of the integration reform. Instead, they were concerned about the fee schedule and the reforms on the payment system [24].

##### Economic growth and stability and financial feasibility

In South Korea, the expansion of the health insurance to the self-employed or workers in the informal sector was a significant challenge for the Universal Coverage scheme. Economic and political factors facilitated the expansion of the insurance to the self-employed, which was the last group that joined the National Health Insurance (NHI). The economic growth of the 1980s improved the financial power of the self-employed to pay for the insurance. At that time, the economic growth increased by 12%. Also, the government had the financial power to subsidize the self-employed [23]. A strong political regime and significant economic growth were effective on the compulsory registration for insurance and cooperation of employers to pay half of the contributions of the employees; and there were few problems related to false reports of wages or evading registration [22, 25]. Other facilitators were mandatory participation and well-established information technology infrastructures. The government made it obligatory for insurers and providers to participate with NHI and all people were obliged to enroll in the national scheme and information technology made the following activities possible which are necessary for merging and extending the health insurance including eligible criteria management, benefit management, claims review etc. [25].

#### The resistances and limitations ahead of the merger

##### Request by rural population for more investments in health infrastructures in rural and remote areas

In order to merge HIFs and create a national health insurance to provide equal benefit packages for all, it may be necessary for the government to make greater investments and provide more financial support to develop infrastructures in rural and remote areas for the underprivileged groups. In Korea, unlike the industrial workers, the extension of insurance to the self-employed faced tough resistance. Farmers wanted a rise in government subsidies and development of health centers in rural areas to improve their access. The government was forced to comply. At first, the government subsidy to the self-employed was about half of the total revenue of the self-employed insurance fund. In Korea, despite high migration rates from rural areas to urban areas, they did not face any serious challenge in this regard as benefit packages of different schemes were the same even before the merger of HIFs [22].

##### Concerns of workers and employers about increase in contribution rates

In Korea, the employers, large corporations which paid half of the contribution of the employees and those with high incomes and low risks were among the potential opponents, as they were concerned that due to the problem of income assessment in the self-employed, the unified insurance system would place more pressure on industrial employers and workers to pay their contribution [22]. The company-based health societies which provided exclusive benefits for their employees were against integration as they believed that integration would lead to eliminating the company-based health societies and in turn the company-based welfare programs [24, 26].

##### Concerns about identifying and setting rational contribution rates for informal workers

Also, in countries that a large part of the population is active in the informal sector, there are problems related to identifying and collecting contributions of the self-employed, part-time workers, and seasonal workers [27]. In Korea, the problem of identifying the income and contribution of the self-employed was the main problem to integrate financial contributions of employees and the self-employed [28].

##### Opposition expressed by trade union concerning about downsizing

In Korea, the structural change and reduction of personnel was one of the intended goals of National Health Insurance system which was faced with the strong opposition by the trade union that was the representative of its employees. This opposition was a serious barrier to achieve expected reduction in the number of personnel and overall administrative costs [13].

#### Positive consequences of the merger

It is worth to specify that what kind of problems can be solved in the health system and health financing area as a result of merging HIFs. Also, the clear explanation of potential achievements can be effective on supporting the implementation of the program and reducing the resistances by opposing actors. In South Korea, the following benefits can be mentioned as the consequences of consolidation:

##### Improving health financial equity

Combination of health insurance schemes, changing financial flows, and increase of the power of health insurance system as a result of combination, can significantly and positively improve the equity in health financing. For instance, in South Korea, the merger of HIFs for the self-employed created the same contribution rates for all the self-employed across the country. The National Health Insurance considered some discounts for under-privileged groups based on their income and geographical location. 62% of the households were paying a lower contribution compared to the times before the merger while the residents of one of the richest counties in Seoul paid greater contribution (36.3% increase in average rate) than before the merger. Therefore, the merger had improved the equity in financing among the self-employed society [13].

On the other hand, the uniform system had improved the contribution equity among the industrial workers. The equalization of the contribution rates after the merger of the industrial workers HIFs showed that 56% of the insured pay a lower contribution. The contribution of the employees with more than 1300 US dollars’ income increased. This meant that the more they earned the higher contribution they paid. The amount of changes in contribution differed based on the number of employees. The contribution of the employees working in the firms with less than 10 employees was reduced by 17% while the contribution of the employees of firms with more than 1,000 employees increased by 19.4% [13].

##### Reducing the administrative costs

In Korea, many HIFs did not have economies of scale due to their small size, and it seemed that the merger would reduce the administrative costs. Before the merger, the administrative costs were 4.8% for the Health Insurance Scheme of the government and school employees (the single insurance), and 9.5% for the insurance scheme of the self-employed. In 2004, the administrative costs of the National Health Insurance were reduced to 4% of the total costs [22]. In Korea, after the merger of the regional HIFs of the self-employed with the HIFs of government employees and school teachers in 1998, 227 HIFs of the self-employed and 19 HIFs of the government employees were reduced to 162 regional HIFs, and the number of personnel was reduced from 10,849 to 9073 in December 1999 [13].

#### Probable risk and negative consequences of the merger 

##### Risk of moral hazard and catastrophe of shared resources

According to the experience of South Korea, the single payer system might face moral hazards. Before the merger, each HIF is responsible for its own financial outcomes, and the insured are interested in the financial saving and keeping their own HIF financially sustainable. However, after the merger, they may be less concerned about controlling health expenditures, and health service utilization may increase. As the financial resources are shared with the whole population, collection of the contributions from the self-employed may not be done as actively as before [13].

##### Risk of facing more political resistance and less flexibility in making health insurance decisions at the national level

After the merger and under a single insurance system, major decisions on the health insurance, such as adapting premiums and benefit packages, will become national issues rather than being a local concern. For instance, the process of adjusting contributions to cope with increasing health costs will mostly be political and less flexible. However, the single insurance system can provide an opportunity to highlight some health insurance issues at national level, issues which may be kept neglected in the fragmented health insurance system [13].

##### Need for more financial support by the government

One issue that must be considered while merging HIFs is that the long-term financing of the single HIF must be guaranteed. In South Korea, the National Health Insurance program experienced financial instabilities. Those opposing the merger claim that moral hazards and inflexibility in increasing the contributions after the merger have caused financial crises. In most countries, government engage financially only in some public schemes or for some groups of population, while after merging, the single national scheme needs more financial support by the government as the financial instability of the single insurance system requires intervention by the government [13].

#### Other contributing health reforms alongside the merger

##### Need for moving towards close-ended payment methods

It is worth mentioning that South Korea policy makers have tried to applied close-ended payment methods in order to increase efficiency, reduce health care expenditures and make the use of health care services more reasonable. In South Korea, since the beginning of the National Health System, fee-for-service payment has been used to reimburse the providers, which has led to an increase in the volume of health services utilization. In order to change the fee-for-service system into a prospective Diagnosis Related Group (DRG) system, a new system was implemented voluntarily as a pilot study in some health care institutes. The results were positive, and it had positive effects on the behavior of the providers. However, due to the oppositions of providers, the new system was not expanded to all providers. Currently, changing the payment system into DRG or capitation is one of the main challenges of the health insurance system [22]. To increase the financial stability of the national scheme, emergency and accident health services are reimbursed by a separate central health insurance fund [29].

#### Future challenges

Despite admirable progress in extending population coverage by creating NHI, it has not been so successful to improve some of health financial indicators according to the three aspects of universal health coverage. Out-of-pocket (OOP) expenditures have been high compared to the OECD countries and it was 36.8% in 2015 while the average was 19.5% in OECD. The performance of NHI has not been satisfactory in terms of extending health services coverage and financial protection which has imposed high pressure on the underprivileged groups. This caused the Korean Government to introduce a NHI reform in August 2017 known as “Moon Jae-in Care” to improve NHI by including all uninsured services into the insurance benefit package [25].

### Turkey

#### Health insurance system before and after the merger 

There were 5 HIFs in Turkey before the merger as follows: 1- The Social Insurance Organization (SIO) which provided health services and pension for employees of the private sector, blue collar workers of the public sector and agricultural workers as well as their dependents, and it covered 47.91% of the entire population in 2007. 2- Bag-Kur or the Social Insurance Agency for Merchants, Artisans and the Self-employed which covered the self-employed, comprising 22.5% of the population. This scheme does not directly provide medical services, but purchases the required services through contracting with public and some private health sector. 3- The Government Employees Retirement Fund (GERF) which was a combination of health insurance and pension fund covered 15% of the population in 2007. This fund covered in-patient and outpatient services. Similar to the Bag-Kur, this fund did not have medical centers of its own and purchased services from the public or private sector. 4- The Government Employees Insurance Fund 5- The Green Card Program: In 1992, the government introduced the Green Card Program for helping the poor who could not pay for in-patient health services [30–32]. The number of people having the Green Card was more than 14 million in 2007. More information about the Turkey health insurance system has been shown in Table 3.

#### Policy objectives: reasons behind moving toward merging of HIFs

The health financing system of Turkey experienced serious problems in the late 1990s and early 2000s. The first issue was related to insufficient and inequitable financing in the health system. Until 1990, nearly 3.8% of the GDP was allocated to the health system, which was much lower than the OECD and other European countries with similar revenue (7.4%). Low health costs had combined with an unjust and fragmented insurance system. The five existing insurance schemes had various services packages and different contractual arrangements with health care service providers, which had led to inefficiency and inequity in the health system. Despite being insured, some people had difficulty in accessing services due to a sever lack of human resources in the health sector. Different contribution rates, benefit packages with different range and depth of services, various access rules and privileges among different insurance schemes along with organizational fragmentation had created significant disparities in the quality and accessibility of insurance services. This led to structural reform of health insurance system in Turkey as a masterpiece of the Health Transformation Plan in 2003 [31].

#### Process of the merger

In 2006, a single system titled “the General Health Insurance Scheme (GHIS)” was established within the Ministry of Labor and Social Security, to merge all existing HIFs including the Social Insurance Organization, the Social Insurance Agency of Merchants, Artisans and the Self-Employed, and the Government Employees Retirement Fund under one umbrella, “Social Security Institution” [31, 33–35].

In 2006, the Grand Assembly ratified the Social Insurance and the General Health Insurance Law to bring together the five health insurance schemes within a unified General Health Insurance scheme. The Turkish Medical Association, medical professionals unions and the Republican People’s Party opposed the law in the constitutional court. In light of pending presidential and general elections, the government postponed the enforcement of the law until October 1, 2008. Before implementation of the law in 2008, it was modified three times and three schemes including the Social Insurance Organisation, Bağ-Kur, and the Government Employees Retirement Fund were transferred to the Social Security Institution. In the next step, the Active Civil Servants Health Insurance Scheme in January 2010 and the Green Card scheme in 2012 were transferred to the Social Security Institution respectively [11, 31, 36].

Through the Health Transformation Program (HTP) in Turkey, synchronizing the health benefits and coverage across the different health insurance schemes including Green Card began even before establishment of the Social Security Institution. In 2005, Green Card holders were given access to outpatient care and pharmaceuticals and Social Insurance Organization beneficiaries were given access to all public hospitals and pharmacies [31]. In 2006, the pharmaceutical positive list was integrated across all health insurance schemes, including that of Green Card holders. In 2007, legislative measures mandated that all Turkish citizens would have access to free primary care, even if were not covered under the social security system. Under the Health Implementation Decree of 2007 (*Resmi Gazete*, 2007), benefits across the formal health insurance schemes were further harmonized [36]. The enforcement of the GHIS law in October 2008 has completed the harmonization of the benefits package. In 2007, a new electronic information management system called MEDULA was introduced to standardize and unify the process of claim and utilization management across all HIFs [31].

#### Facilitators of the merger

##### Leadership and political commitment

In Turkey, the political stability which was achieved by the Turkish government and enjoyed the support of the majority of the Grand National Assembly was an important factor in health reforms and evolution. The Grand National Assembly managed to pass the laws which were proposed by the government, unlike the federal governments which were unable to implement policies for many years. A committed transformation team in the ministry of health, prime minister’s firm support of reform, leadership and persistence of the minister of health, and great management created the necessary conditions for implementing the laws which were passed by the Grand National Assembly. This team, which enjoyed the support of the prime minister and the cabinet, operated constantly for more than 10 years [11].

##### Formation of a research team, training workshop and executive program

In Turkey too, the main reason of success of HTP and universal health coverage (UHC) was the transformation team – very committed transformation team which consisted of people working together for 10 years from 2003 to 2013. The transformation team had an active role in understanding, designing, implementing and monitoring the HTP, and provided strategic orientation, continuity, and institutional memory for transformation. The team worked with international agencies and experts, and provided the connection between strategic and operational phases of the implementation. Regular surveys of field conditions formed strong connection channels between the provincial leadership, local implementation teams, and the Ministry of Public Health. Another factor in the success of the HTP was the speed of policy implementation. As soon as a decision was made or a law was passed, it was implemented according to the time table which was monitored weekly by the transformation team. Whenever there was a delay, the implementation strategies were changed and local groups or special teams of the Ministry of Public Health were responsible to identify and solve the challenges. These quick implementations prevented organized resistance against reforms, and helped overcoming bureaucratic resistances. The speed of implementation was legitimized by a quick explanation of benefits for users and the public [11].

##### Informing people, focusing on consumer satisfaction and acceptance of the change by the people

In Turkey, the transformation team assessed the acceptability of changes made by HTP across different groups of people, through establishing focus groups and analyzing beneficiaries. The findings of the focus groups and beneficiary analyses were used to redefine the range of HTP, inform the public, and speed up the process of implementing the program. Beside the focus group researches and stakeholder analyses, annual household surveys were conducted by the Turkish Statistical Institute. The surveys introduced an index for measuring the satisfaction of people and their reaction to the reforms announced by different ministries [11].

##### Economic growth and stability and financial feasibility

Like South Korea, in Turkey, the economic stability and fast growth of GDP, which occurred between 2003 and 2012, provided the required financial capacity, enabling the government to invest in social affairs. As economic growth increased, the government increased the health budget and investment in the health sector. Also, the investments of the private sector in health were increasing. Along with the stable growth of GDP, new laws and methods improved tax collection, and balanced economic policies increased tax collection and decreased inflation and unemployment rates. Increased tax revenues of the government, encouraging privatization and foreign investments enabled the government to expand the coverage of the Green Card using the public budget revenues and establish the universal public health insurance [11].

##### A comprehensive strategy based on evidence

The HTP scheme in Turkey was devised based on the evidence and experience of other countries such as Belgium, Cuba, Denmark, Estonia, Finland, Mexico, Thailand, and England. The Ministry of Health successfully benefited from the cooperation of international agencies and national and international experts. Along with international experience, local experience and studies related to the coverage and efficiency of the health sector and obstacles ahead of the health system of Turkey were used.

Other factors that facilitated the merger of insurances as a part of the Health Transformation Plan in Turkey included creating a receptive environment, considering health as the fundamental right of people, constant learning and monitoring of the programs, and flexible implementation [11].

#### Resistances and limitations ahead of the merger

##### Concerns about inconsistency between merger law and national laws such as constitutions

In Turkey, although the law of the General Health Insurance Scheme (GHIS) was approved by the Turkish Grand National Assembly on May 31, 2006, and it was expected to be implemented on January 1, 2007, the opposing party (the Republican People’s Party) claimed that the law was against the constitution. This law faced the opposition of the Turkish Medical Association and other professional medical unions, and was challenged in the constitutional court. The law was revised three times before its implementation. The government postponed the enactment date until July 1, 2007 in order to make the necessary changes. In the light of the presidential and general elections, politicians convinced the government to further postpone the implementation until January 1, 2008. The final revised version of the law was enacted on April 17, 2008 and implemented on October 1, 2008 [35].

#### Positive consequences of the merger

##### Improving equity in health financial and health care utilization

Merging has been successful in improving health financing equity as, the share of out-of-pocket health expenditures in Turkey was 19% of the total health costs 3 years after the implementation of the program, which was considered fairly low [37]. By implementing GHIS nearly all population are now under health insurance coverage. Studies show that introduction of GHIS has improved both financial protection against high health expenditures, and equity in access to health care across the population and has made health financing more progressive as richer households pay more of their spending for the health than poorer families. The studies also indicate highest reduction in out-of-pocket spending in Turkey among OECD countries between 2000 and 2012 and reducing OOP payments among the poor as a result of extending Green Card Coverage [30, 31, 38, 39].

##### Creating a single information bank and removing the coverage duplication

Before the General Health Insurance Scheme (GHIS) in Turkey, different statistics were presented by different information system for health insurance population coverage statistics, ranging from 67.2 to 84.5%. In one case 101.15% was reported. These differences were result of lack of single information system. The Organization for Economic Co-operation and Development of Turkey reported that many people have more than one insurance policy, which leads to duplication and an increase in the number of the insured records [35].

#### Probable risk and negative consequences of the merger

##### Need for more financial support by the government

One issue that must be considered while merging HIFs is that the long-term financing of the single fund must be guaranteed. In Turkey, the health system’s share of Gross domestic product (GDP) quickly increased form 5.4% in 2000 to 6.7% in 2009. This number was constant in 2010 and 2011 [11]. Critics expressed their concerns about the probability of increasing the financial engagement of the government by implementing universal health coverage as they believed that unemployment makes it difficult to collect premiums and the government have to pay instead of them [32].

#### Other contributing health reforms alongside the merger

In Turkey, alongside merging HIFs, new roles were defined for the Ministry of Health to enhance the stewardship role, and also new executive (operational) responsibilities were assigned to new organizations. Between 2003 and 2010, the Ministry of Health was responsible for the management of the hospitals affiliated with the Social Security Institution and also the Green Card Program. Also, after the announcement of the law of the reorganization of the Ministry of Health and the autonomy of hospitals, the ministry of health focused on devising strategies and policies, assessing the performance of the health system, and monitoring the responsiveness and inter-sectoral coordination. The executive responsibilities related to public health, concluding contracts, providing health services, and assessing health technologies were assigned to new quasi-governmental organizations. After the introduction of the uniform public health insurance, the Social Security Institution took over the management of the Green Card Program [11].

Another structural reform was the transformation of the hospitals of Turkey. Public hospitals such as hospitals of the Social Security Organization which were not being managed by universities or military organizations were transferred to the Ministry of Health in 2005 under the Health Transformation Program (HTP). The apparent reason for this action was to ensure uniformity among public health providers in terms of service quality; however, the subtle motivation was to improve the process of transferring hospitals to local governments which was considered within the framework of public administration reforms [35].

Along with other reforms, the health transformation plan conducted four new key programs in the human resources department in order to emphasize the problems related to the shortage of human resources in the health system. The first program was implemented to increase the number of schools and colleges for medicine, nursing, midwifery and other related fields. The second program included a raise in wages and incentives based on performance for hospitals and primary health care centers, and the possibility for constant salary rise for healthcare employees. The third program focused on writing new contracts with the health employees and purchasing services from the private sector. And finally, the fourth program was the law of prohibiting dual practice for physicians, which was passed in 2010. Based on this law, the physicians who were not employed by the Ministry of Health had to work full time in government hospitals. Also, they were banned from working in the private sector at the same time [35].

##### Emphasizing on primary health care services

In 2005, the health transformation plan in Turkey introduced a family physician-centered primary care model and emphasized on increasing resources in three areas – physical resources, human resources, and creating capacity for human resources. Based on this model, each family physician provided services more than what was provided in rural health center or health centers, to a population of 4000 people. After 2005, about 20,000 family physician teams were formed. The existing infrastructures were improved and most rural health centers and health centers were kept or merged with family physician centers. Until 2011, nearly 6250 new family physician centers were founded [11].

#### Future challenges

In Turkey the main current challenge is keeping the universal health insurance financially sustainable as the health expenditures are going up due to the comprehensive scope of Turkey’s UHI, changing demographics and various economic indicators So it is said that Turkey should address two policies, finding new financial resources and focusing on cost containing measures. Identifying the informal sector workers who do not report their real income or those workers that have not registered in the labor force and levy tax on them have been mentioned as potential solutions to generate new financial resources. The following measures have been advised to curb the total health care expenditures: narrowing the benefit package under coverage of GHIS as it is considered so extensive, moving towards bundled payment methods like DRG, updating and contracting the list of pharmaceuticals, setting new copayments and coinsurance rates, emphasizing on primary health care and introducing gate keeping role and implementing referral system into family physician program to manage the flow of patients through the health system, and forcing the public hospitals to be autonomous and manage themselves on their own [31].

### Thailand

#### Health insurance system in Thailand before and after the merger 

In Thailand, the structure of insurances before the merger was as follows: The Medical Welfare Scheme: It was a free program covering the elderly (older than 60) and children under 12, the disabled, monks, religious leaders, and veterans. Also, there was the Civil Servant Medical Benefit Scheme (CSMBS) which covered government employees, retirees, and their family members. This scheme is considered the most generous insurance scheme in the country. The other scheme is the Social Security Scheme (SSS) which was established to cover the illnesses and injuries of the private sector employees (but not their dependents). The Voluntary Health Card Scheme (VHCS) was the continuance of a community financing initiative of 1983 which started as a scheme for covering the workers of the informal sectors [29, 40–42]. More information about Thailand health insurance system has been provided in Table 4.

#### Policy objectives: reasons behind moving toward merging of HIFs

In Thailand, each health insurance scheme had its own specific rules, regulations and benefit packages for its own beneficiaries. The uninsured had to pay high prices in governmental health centers or choose private centers. A low number of people had private insurance. These HIFs were different in terms of size of population overage, the definition of eligibility, benefit package, payment method, financial resources, per capita expenditures, and subsidy. Each of these schemes covered different parts of the population and had different methods of reimbursement and gaining revenue. Improper and inequitable distribution of government subsidies across the aforementioned insurance schemes had extremely affected the productivity level and benefits of each scheme [43, 44]. About 70% of the population was under the coverage of the four public health insurance schemes, while private insurance played almost no role. The remaining 30%, more than 15 million people, were not covered by any medical insurance and had to pay out-of-pocket for health care services and medicine [29, 41]. Inefficiency was prominent in almost all of the schemes due to various reasons such as adverse selection, moral hazards, and allocative inefficiency [29]. All of these problems made the Thailand government to merge health insurance schemes in 2002 as a solution to solve above challenges.

#### Process of the merger

The three main insurance schemes of Thailand after merging are the Universal Coverage scheme, the CSMBS under the administration of the ministry of Finance, and the Social Security scheme under the administration of the Social Security Organization. The Universal Coverage scheme covered all the uninsured, voluntary health card holders, and those covered by the medical welfare scheme under a single unified program [29, 41].

#### Facilitators of the merger

##### Leadership and political commitment

In Thailand, universal coverage was considered as an important national issue by the Thai Rak – a new political party created by the richest capitalist in Thailand, Thaksin Shinawa. This scheme was a key part of this party for the election of 2001. The leaders of this party considered a policy for achieving universal coverage due to its intrinsic popularity, financial and technical feasibility according to the studies [29].

##### Formation of a research team, training workshop and executive program

In Thailand, the Working Committee on Universal Coverage was formed in 2000 with the support from the Health Systems Research Institute. After winning the election, the universal coverage policy was included in the government’s policy and immediately, a workshop on the issue of implementing universal coverage was organized by the prime minister. Also, a task force was established for the purpose of the implementation of the universal coverage program, and the Ministry of Public Health was tasked with the implementation of the program. With several members of the Labor Committee present in this workshop and in the task force, the program was implemented exactly according to the recommendations of the committee members [29].

##### Informing people, focusing on consumer satisfaction and acceptance of the change by the people

In Thailand, the media played an effective role in keeping people informed, and was active in informing people of different subjects of the universal coverage and consequently increasing people’s awareness [29].

##### Economic growth and stability and financial feasibility

In Thailand, in accordance with the spirit of the 1997 constitution, the Universal Coverage Committee was established in 2000 to assess the potential alternatives for the universal coverage. This committee studied and confirmed the financial feasibility of achieving universal coverage and stated that assuming an efficient insurance system, it can be affordable. In total, 100 billion B was predicted for the universal coverage per year, which was not much higher than the 76 billion B which was spent annually by different health insurance schemes and the public health budget at that time [29]. Thailand committed itself to expand coverage under the Universal Coverage Scheme (UCS) in 2002, after the Asian financial crisis when macroeconomic growth prospects were still fragile. However, economic growth has been one of the important enabling factors underpinning the subsequent expansion of UHC in many countries once they adopted UHC [14].

##### Comprehensive information system

In Thailand, the first problem was to identify the uninsured (those who were not covered by SSS or CSMBS) considering the fact that there was no database of CSMBS beneficiaries available. Consequently, a comprehensive information system was quickly established using a government registration database to avoid the duplication of health insurance benefits. This was not an easy task since more than 50 million records had to be created [29]. Reliable information system in place made the following activities easier which are vital for reaching UHC: assigning a unique citizen ID number to each citizen, identifying members of the UCS and register them with a preferred provider network, dynamic and automatic transferring of members between the three public insurance schemes by changing their eligibility criteria [41].

#### Resistance and limitations ahead of the merger

##### Opposition by population groups enjoying better benefit package

One of the main challenges and resistances facing of the merger of HIFs is the objection of HIFs with more generous packages, especially when the single insurance is affected by the low performance in the past, and having low quality benefit packages due to insufficient premium level [27]. In many countries, there are different schemes for government employees, private sector staff, and the self-employed, and each has its specific benefit package. For instance, in Thailand, government employees use a more generous benefit package. Equalization of benefit packages for all HIFs will face oppositions from stronger HIFs [22]. In Thailand, the labor unions were concerned about the transferring of budget from the Social Security Fund to the Universal Coverage scheme, and considered the benefits of the Universal Coverage to be lower than what they already had. The unions organized several street protests to demonstrate their concerns against the merger of the Social Security scheme with the Universal Coverage Scheme [29].

Using public hospitals and health care centers by the members of health insurance organizations with poor benefit packages and low financial resources can cause distrust among those who are under coverage of health insurance organization with generous benefit package and enjoy high quality health services in private sector . Private employees may think that the single insurance provides low-quality health care which is currently provided for the members of weak insurance schemes. In order to solve this problem, it is better that the single insurance scheme focus on those who are not under coverage of any HIFs fist. Gradually, the compulsory insurance fund should improve the quality of its services while simultaneously proving that it is possible to provide high-quality services with lower prices and contribution rates than private health insurance [27].

The differences in benefit packages across health insurance funds will lead to social classification and in turn portability problems between various funds, especially when migration rates from rural areas to urban areas are high.

##### Concerns of workers and employers about increase in contribution rates

In Thailand, a strong opposition was formed by the members of the Social Security Scheme (SSS), especially labor unions, against the expansion of SSS to other groups, because they feared that the SSS’s resources would be used as subsidy for the rest of the population. The Social Security Office was concerned about the actuarial feasibility and the limited support from the employers [45].

In Thailand, the Medical Association supported the Universal Coverage Bill but showed opposition to the medical responsibility clause included in the bill. The Medical Association was concerned about the increase in the purchasing power of single national insurance scheme [11].

##### Concerns about the monopsonistic purchaser power of NHSO

Providers were against applying bundled payment methods such as capitation and diagnostic-related groups under global budget something that it is hoped to be implemented by having a strong insurance scheme. Strategic purchasing of the NHSO was not welcomed both with hospitals and pharmaceutical companies as hospital preferred to purchases medical devices on their own and pharmaceutical companies could earn higher benefits by selling directly to hospitals rather than to the NHSO [41].

#### Positive consequences of the merger

##### Reaching universal health coverage and leaving no one behind

Normally, one of the consequences of the multiple HIFs is that despite existing different health insurance organizations alongside each other, a part of the populations is not covered by any insurance for different reasons. Achieving universal health coverage can be considered as one of the advantages of merging HIFs and creating a single national scheme. In 2002, Thailand achieved universal coverage through a strong government with political commitment, supported by civil society, and in 2014, nearly 99% of the population were under coverage.

##### Improving health financing equity

Apart from improving access to outpatient care and hospital admissions especially among elderly people, UCS have been successful in improving financial indicators for instance the household out-of-pocket payments have been reduced from 34% of total health expenditure in 2000 (before the UCS) to 12% of total health expenditure in 2014. It also has reduced chance of facing catastrophic and impoverishing health care expenditures [41].

#### Other contributing health reforms alongside the merger

In Thailand, the wide geographical coverage of health care facilities of the Ministry of Public Health (MOPH) was an important foundation for the implementation of the universal coverage scheme. This meant that the members of the scheme – many of whom were living in rural areas – had access to the health care services. In 1970, Thailand began to build more hospitals and train more nurses and doctors, and as a consequence, the ratio of the population to beds, nurses, and doctors was significantly improved by 1990 [46].

##### Emphasizing on primary health care services

In Thailand, the Universal Coverage scheme, aiming to provide equal access to health care for all, has three defined characteristics focusing on the constraining total health care expenditures: UHC scheme is financed by taxation providing free-of-charge services, a comprehensive service package focusing on primary health care such as prevention of illnesses and improving the health status, and using fixed capped budget to reimburse provider [46].

In Thailand, the Universal Coverage program is trying to use financial mechanisms to improve primary health care. The main financial resource of the Universal Coverage scheme is the taxation revenue which is paid annually by the government per person (1899 baht per person in 2007) [28, 29]. This amount is directly paid to the providers that have a contract as per capita [40, 47].

##### The purchaser-provider split

One of the significant innovations implemented on a parallel with introducing UHC scheme was the establishment of the National Health Security Office (NHSO) in Thailand to act as a purchasing agency for the beneficiaries of the Universal Coverage scheme. In fact, the purpose was to separate the purchaser from the provider. This meant that the Ministry of Public Health will not have much control over the government expenses related to the public health services [46].

The NHSO was established as a quasi-government organization with the minister of public health as the head of its executive board. Operation and management of the Universal Coverage scheme fund was transferred from the Ministry of Health to the NHSO as the purchaser of health care for the members of the Universal Coverage Scheme [29].

##### Need for moving towards close-ended payment methods

In Thailand another significant reform was change of the payment method. In the UHC insurance scheme, payment for outpatient services is in form of capitation and the government pays this capitation to the contracted centers, based on the number of people who are registered in these centers. The amount of this capitation is calculated annually. For hospital services, the employed method is based on diagnostic-related groups. Also, prevention and health promotion services are included in the basic package, and the method of payment to providers is based on the capitation system along with the performance-based payment [48]. This made it easier to project the total funding needs to manage the UHC insurance and assessment of financial feasibility [41].

#### Future challenges

The harmonization of the three government health insurance schemes is still the focal point of debate after the beginning of the universal coverage in Thailand. Inequality between these three schemes has challenged the right to public health care as the right to access health care with equal standards for all the people of Thailand. Recent studies showed that harmonization between UCS and social health insurance has been achieved in terms of benefit package and applying closed-end provider payment methods but there are still problems with CSMBS. CSMBS uses fee-for-service for out-patient care and uses different bands of DRG payments for in-patient services in a way which favor tertiary and teaching hospital over other hospital leading to quadruple per capita expenditures in CSMBS compared to UCS [41, 45, 46].

### Indonesia

#### Health insurance system in Indonesia before and after the merger

In Indonesia, the structure of insurance included the following organization before the merger: 1- Civil Servant Social Health Insurance Scheme (Askes), which covered all government employees and retirees and military personnel. 2- The Private Employee Social Health Insurance Scheme (Jamsostek). 3- The Commercial Health Insurance scheme JPKM (HMOs), which is entirely similar to the Health Maintenance Organization (HMO) of the US. The JPKM insurance is classified under commercial insurance, and provides in kind benefits which are managed by various managed care organizations that are not insurance companies. Several Social Safety Net schemes have been introduced over recent decades to assure the access of the poor to necessary health services with different level of success. The last program to cover the poor were Jamkesmas and Jamkesda that were provided by national and regional governments respectively [27, 49–51]. Details of the health insurance schemes are presented in Table 5.

#### Policy objectives: reasons behind moving toward merging of HIFs

In Indonesia, the health insurance system was far from just due to improper implementation. For government employees, the payment rates of the social health insurance to hospitals were much lower than governmental medical tariffs. Financial support for the poor and vulnerable groups such as pregnant mothers, children under five and the elderly was seriously insufficient. Different programs to cover the poor suffered from underutilization and could not identify the real poor people precisely and other people than the poor used these programs. Poor people were not able to use cards due to lack of understanding, stigma of being perceived as poor, living in remote areas and lack of public facilities. Therefore, in November 2011, Indonesia enacted an ambitious act which forced the five government HIFs, which had covered different parts of the population, to merge into a universal program in order to reach cross subsidies, reduce administrative costs, providing the same benefit packages for all, and reduce inequities in access to the benefit packages [50].

#### Process of the merger

In November 2011, a new law was passed mandating BPJS, the ‘Social Security Administering Body’. The National Social Security Council was established to address the law. The council created two social security corporations including the Social Security Agency for Health as a non-profit trust fund called BPJS Health, while the workplace insurer JAMSOSTEK was assigned to administer pensions, life and workplace insurance as its new mission called, BPJS Workforce. Assets, liabilities, participants and staff of the corporations were automatically transferred to the new bodies. BPJS Health began to operate in January 2014 and integrated the previous existing HIFs including Askes, Jamsostek, Jamkesmas, Jamkesda and also military personnel (Asabri). The premiums for the poor would continue to be paid by the state. BPJS would operate as a single quasi-government entity. It uses a prospective payment methods including capitation for primary care providers and Diagnosis Related group (DRG) on the basis of Indonesian case-based groups for secondary health providers. It is worth mention that participating in the BPJS is mandatory and employers are not allowed to opt out of BPJS [51–53].

According to the Roadmap of National Health Insurance, it is supposed that all Indonesian citizens will be covered by the BPJS Health by January 2019 [51]. By October 2018, 203 million people forming 75.88% of the whole population were covered by BPJS [53].

#### Facilitators of the merger

##### Leadership and political commitment

In 1999, Indonesians elected PDI-P party led by Megawati Sukarnoputri who had campaigned to work on equity. In 2000, the new parliament included the “right to receive medical services” in the 1945 constitution and again later on the state was obliged by the constitution to ensure providing health service as well as developing a social security system for all citizens. PDI-P leader Megawati, then Vice-President assigned a working group of over 60 people to draft the social security law. In its first version, the integration of all four state-owned insurance firms into a single entity was specified to operate as a single payer, not-for-profit trust fund. According to the reports, the bill of social security reform was revised 56 times before submitting to the parliament in January 2004 [52, 53]. Although by changing the President and taking the office by the next President, Yudhoyono, policy agenda changed and social security reform was put aside for the next 10 years until it was came into effect in 2014 [53].

#### Resistance and limitations ahead of the merger

##### Concerns of workers and employers about increase in contribution rates

In Indonesia, the private sector employers may refuse to join the National Health Insurance as they are afraid that the contribution rates might increase partly because they could no longer opt out of the state-run scheme. They also argued that the mandatory schemes violated human rights. Also the current competitive market may oppose formation of any kind of mandatory health insurance [27, 52]. In, Indonesia, during the passing of the law in the parliament, strong resistance was formed by labor unions and employers’ organizations [54]. Also, the Ministry of Labor was against the unified insurance bill. Initially, the Ministry believed that the bill would put more pressure on the workers and that the Ministry might lose control over the social security funds of private employees. Under the social security system before the merger, government employees paid all the contribution themselves, while private sector employees did not make any contribution. The National Health Insurance Bill (NHIB) proposed a shared contribution between employers (government as the employer of government employees) and employees. The private sector employees who did not have any contribution until then, were strongly against the bill. The minister of labor threatened to veto the bill if the shared contribution was approved by the parliament. Even employers’ organizations, business offices, and several government authorities rejected the mandatory essence of the Social Security. Employers were concerned about the amount of contribution which would be specified for them. They had some estimation based on their calculations, and opposed bill since they concerned about the probable financial problems that might be imposed by the bill [52, 54].

##### Concerns about sharing benefit package and premiums with underprivileged groups

Employers worried that premiums paid by workers and employers would be used to subsidize services for the poor and unwaged, leading to a cut in benefits for those in work [54].

##### Concerns about losing autonomy and getting exposed to transparency

Directors of state firms were reluctant to lose their control on their own cash flows and also worried that the restructuring the health insurance system would open their private profiles to public scrutiny [54].

##### Need for more financial support by the government

In Indonesia, the obligation of government as an employer to pay 3% contribution compared to the currently-paid 0.5%, will require an additional cost of 1.3 trillion rupees annually. Also, obliging the central and local government to pay contribution for the poor will require 5–8 trillion rupees from the central and local budget. The existing financial problems of the government might postpone the coverage of the poor [27].

#### Positive consequences of the merger

##### Reducing the administrative costs

Bigger HIFs will improve the economies of scale, which will in turn maximize the benefits provided for the members. It is estimated that in Indonesia, by implementing the National Health Insurance, similar to the schemes in Taiwan, the Medicare in the US, the Medicare in Australia, the National Health Insurance in Korea and the Philippines, the administrative costs will be reduced to lower than 5%, and can be even further reduced to 3% of the total costs [27].

##### Equity in benefit package and health care utilization

The single insurance system can enhance insurance packages and extend the coverage in favor of the poor and the members of weaker insurances. For instance, it is expected that in Indonesia, the uniform service package for civil servants and the private sector employees will create better clarity, equity, and understanding of the package for providers and members [27].

##### Creating a single information bank and removing the coverage duplication

The single insurance system will lead to the concentration of the statistics and information of beneficiaries. In the National Health Insurance of Indonesia, information system and social security number will be unified across all social security schemes using a unique social security number. The uniform information system will reduce the duplication of coverage and membership, and will lead to a higher efficiency and easier transfer of benefits with changing the job in the dynamic labor market [27].

##### Paving the way for implementing other contributing health reforms

Moreover, merging and reducing the number of HIFs can create a window of opportunity for implementing extra reforms in the health system. These reforms were less likely to be implemented before due to the multiple insurance schemes working alongside each other. For instance, in Indonesia it is expected that creating a national health insurance scheme with higher number of beneficiaries and higher power of negotiation with the providers increase the chances of implementing per capita payment or other prospective payment methods like DRG. These prospective methods will improve the efficiency of the system. Also, another reform which leads to a higher efficiency of the health system is the better implementation of the family physician program and the referral system. An increase in the number of the insured provides the possibility of using a gatekeeper system and improves the utilization of the family physician as the gatekeeper [27]. One of the challenges which exists in most of health systems is the unbalanced distribution of health work forces and concentration of health care providers in big cities. Pooling of HIFs will improve the equality of the redistribution of providers in all areas. Under a single fund, the money will follow the patient. Currently, about 25% of all physicians in Indonesia are living and working in Jakarta (Providing services for nearly 8% of the population) [27].

#### Future challenges

According to the studies the five major challenges ahead of implementing the BPJS are as follows: the fragmented health financing system, decentralisation, demographic transition, high out-of-pocket spending, and low levels of spending on health by the central government [50, 55]. Decentralization of government in Indonesia began in 2011. There are four levels of government including national, provincial, district and sub-district level which have made health financing reform more complicated. There are two parallel programs for covering the poor, the Jamkesda at national level and Jamkesmas which is managed by the regional governments. Jamkesmas began to operate as a part of poor people were not categorized as poor based on the national criteria. Apart from these parallel programs, applying to the national health insurance scheme and running the regional health insurance schemes were problematic for regional governments and also confusing for both providers and patients. Also local governments were obliged to implement all services communicated by the mandatory universal health insurance which is not feasible in some cases [50]. Membership is another challenge of the National Health Insurance System (NHIS). Poor people who are paid by the government dominated the NHIS forming almost 60% of membership in 2017 which means it put heavy pressure on public budget [53].

The second challenge related to the membership is known as “the missing middle for the NHIS”. This problem refers to people who work in the informal sector or those who are not living in poverty but are not covered by the NHIS because of low self-enrolment. This population group is typically unwilling to participate in insurance schemes. As this group formed approximately 60% of Indonesia’s labour force in 2014, low enrolment by this group lingers reaching to universal health coverage and also jeopardize long term financial stability of the NHIS. These problems alongside low coverage of services, high out-of-pocket expenditures 48·3% of total health expenditures, low average contribution per capita $30 in 2016 compared to the average per capita of THE ($129), budget deficit means government should inject more public budget into the NHIS and reliable solutions should be advised.

To overcome financial issues affecting overall UHC sustainability, four policy options have been suggested: first, increase fees for contributing members, given that current contributions are lower than the costs of medical treatment; second, embrace cost-containment measures, such as soft caps on service volumes; third, improve the health-care reimbursement process by more rigorous medical claim reviews; and fourth, promote efficiency of the NHIS.

Finally there is need to develop an affordable and appropriate benefits package, expand service provision and encourage the regular payment of premiums by the non-poor, around half of whom do not currently contribute as the law requires [52].

## Discussion

### The role of country context and factors influencing moving towards merging HIFs

Different countries may decide to merge their HIFs based on different reasons and contextual factors. The experience of studied countries show that differences in benefit packages with different range and depth of services, inequity in paying contribution rates and also inequity in access to health care services between different groups of population were commonly among the main reasons caused the policy makers to merge HIFs. Before merging HIFs, in South Korea, many of HIFs were small in size and were unable to integrate their resources effectively. In Turkey, there were inequities in access to health services between western developed areas and eastern underdeveloped areas, between the rich and the poor, and between urban and rural areas. In Thailand, improper and inequitable distribution of government subsidies across the insurance schemes had extremely affected the benefits of different HIFs and in Indonesia the administrative costs of health insurance funds were high and there were inequities in access to the benefit packages.

The experience of studied countries show that in countries with a fragmented health insurance system, considerable differences among different groups of insured population in the following items including the percent of the whole population under coverage of each health insurance scheme; contribution rates, benefit packages, quality of health care services received by members of different risk pools, and the amount of out-of-pocket payments paid by different groups of beneficiaries for the same health services made officials to put merging the existing HIFs on the agenda to end the health inequities in the health insurance system. Single or multiple risk pools approach is fundamentally influenced by local considerations. For instance the following items can affect moving towards merging and reducing the number of risk pools: is the health system emphasizing on the health equity and efficiency?; is universal health coverage is the priority of health system? and which approach, single or multiple risk pools, can help reaching it in a given country?; the role of government in supporting the health insurance schemes financially; the general socioeconomic condition of the country; financial sustainability and constraining health care services expenditures; the status quo of administration costs of and also the competition status among health insurance schemes; the degree of autonomy or dependency of health insurance schemes to the public subsidies; the degree of rigidity of governmental regulations which the health insurance schemes have to follow to operate such as modification of benefit package or setting premiums; the possibility of redistributing of risks among risk pools without merging them and etc. For instance, in Thailand, the merger began with schemes under Ministry of Public Health including the Medical Welfare Scheme, VHCS, and those without coverage which were financed mainly by the government. According to Thailand’ experience, to start merger it is advisable to begin from schemes which are governmental, depend on governmental fund or operate under one administration such as ministry of health. Where different schemes belong to different ministries, more cooperation and effort in needed to bring all to an agreement for merging.

In South Korea, the contextual factors which facilitated moving towards merging HIFs were as follows: health insurance funds were financed largely by the government subsidy, they were not autonomous, and they were working within tight regulation framework issued by the government. So creating a single national health insurance fund was preferred than dealing with having several HIFs. In contrast, the experience of other countries with different contextual factors such as Germany and the Netherlands indicates why despite having multiple risk pools, merging health insurance schemes is not the only option to boost the performance of health insurance system. Conversely these countries have encouraged competition among their schemes rather than moving towards merging to increase consumer rights to choose among sickness funds and in turn constrain healthcare expenditures. These countries have launched an equalizer mechanism which allocates a risk-adjusted compensation to insurers with higher risk beneficiaries. This system eliminates a part of health inequity among competitive risk funds. In Germany, competition is preferred than merging as sickness funds don’t rely on government subsidy and are autonomous in implementation and government [17, 56, 57].

### Potential advantages and disadvantages of merging HIFs

The experience of studied countries showed that health financial equity has been improved as a result of merging HIFs. By merging, horizontal financial equity in South Korea increased as the contribution rates became the same for all self-employed across the country. Vertical financial equity increased as people with lower income and those working in small firms paid lower contribution rates and the rich and those working in large firms paid higher contribution rates compare to the situation before the merger. In Turkey, also merger increased equity in health financing as the rich paid higher contribution rates after the merger and the amount of OOP expenditures decreased in the poorest group of population. Also the merger increased health equity in Turkey by creating a common benefit package for all groups of population especially in terms of outpatient care and pharmaceuticals and also access to the public hospitals and pharmacies. In Thailand the share of OOP expenditures from total health expenditures reduced after the merger.

Reducing administrative costs was another advantage of merging HIFs. In South Korea administrative costs reduced as a result of downsizing and reducing the number of personnel. The same results are expected to manifest in Indonesia as a result of merging HIFs.

In a fragmented context usually a part of population is out of coverage but reaching universal health coverage becomes easier by creation a national single health insurance fund as the number of population without coverage become clear as a result of creating a single data bank and removing duplication in population coverage. In a single payer system having a part of population out of health insurance coverage becomes less acceptable politically.

The experience of South Korea shows that introducing or changing the policies regarding the health insurance system such as contribution rates and benefit package should be made at the national level which makes it less flexible and more political. This in some cases can affect the national health insurance fund negatively. Bureaucracy and centralized policy making at a national level may result in mismanagement and reducing the speed of decision making. However, some authority and independency should be delegated from national level to the regional administration in some areas like contractual methods and levels of payments to the providers.

The merger of HIFs has the benefit of pooling risks across the country [13] which makes it possible the distribution of cross-subsidies among all groups of population [27].

The international experiences show that the single payer system is more powerful and more efficient in controlling the total health care expenditures [13, 27]. Regarding risk pooling efficiency and financial stability, the single payer is more preferable [22]. In the single insurance system, collection of contributions will be much more efficient than the collection of contributions by different health insurance schemes separately.

Also, the single insurance system will increase the competition among providers, since the single insurance is the only payer and provides a free choice of providers for the people [27].

A single payer may reduce efficiency due to increasing the bureaucracy and decreasing responsiveness; however, in many developing countries with multiple health insurance schemes where people do not have real right to choose between different HIFs, the efficiency lost as a result of merging may not be significant [13]. In a single payer system, the insured do not have the chance of choosing and changing the insurer when the health services are not satisfactory which can lead to dissatisfaction, especially in rich families. However in the single payer system, the free choice of the provider and increased competition among providers can increase the satisfaction of the beneficiaries [27].

### Facilitators and obstacles of merging HIFs

The experience of countries showed that long-lasting financial sustainability especially by the government is a key prerequisite at least to continue and maintain (if not to start) creation of single national scheme for all. The Universal Health Coverage Scheme of Thailand is still facing multiple challenges. The National Health Security Office which manages the universal coverage scheme is currently financed through general taxation, which requires annual negotiations over the per capita budget with the Ministry of Finance. With changes in healthcare technologies and the aging of the population, it is expected that the costs of the program will increase, and the financial stability will be endangered. Therefore, alternative long-term financial mechanisms are necessary [29]. In Indonesia, the International Business Chamber of Commerce, which has representatives from different investor countries, demonstrated its opposition to the National Health Insurance Bill, in several occasions. In their analysis, the USAID consultant stated their concern over the effects of the Bill on the economy of Indonesia and mentioned that if the Bill is going to be implemented, GDP would decrease by 1% [54].

The findings showed that the financial stability, economic growth and also financial support of government are necessary to implement merging and create a single insurance. By creating a single national fund the financial responsibility of the government become more serious and conspicuous as more financial support needs to cover population without health insurance coverage, increase the share of government from contribution rates on behalf of the poor and underprivileged groups, to enhance the benefit package for all, to support the national insurance scheme in the case of facing budget deficiency, to mandate employers and employees in the private sector to participate in the single national insurance and pay their own share of contribution rate and also to invest in health infrastructures in deprived and remote areas.

Like other health reforms, the findings showed that merging health insurance together is a tough political decision. Many actors may oppose merging based on the interests and advantages they may lose or disadvantages they my face as a result of merging. Those with more generous benefit package may oppose sharing it with other underprivileged groups. Employers and employees enjoying low premium rates may concern about paying more contribution rates and those who work in health insurance organizations or even work agencies may oppose merger as it may jeopardize their job stability. According to the experience of Indonesia, these concerns can be more challenging where the health insurance scheme which is going to be center of merger is weaker than other existing health insurance schemes in terms of benefit package, contribution rates, or even the amount of salary it pays to its employees.

Most countries have gradually extended the universal health coverage. Due to the complex process, the required effort to achieve the support and agreement of the interest groups and the time-span needed for developing the organizational and technical capacities, countries choose a gradual approach toward providing insurance cover for different groups. The experience of countries show that the gradual health coverage development approach leads to the creation of multiple risk pools for different population groups with different levels of coverage. This creates new challenges for the assurance of equal coverage and redistribution of resources across risk pools, and once they are established, it will be politically difficult to merge or integrate them since this means that the privileged organized groups must lose some of their benefits in favor of other population groups [14]. Therefore, moving toward a single insurance and consolidation of HIFs, like any other large-scale reform in the health system, have always faced resistance from interest groups.

As mentioned before, bigger differences among the existing health insurance funds (differences in benefit packages, contribution rates, financial resources, number of beneficiaries, out-of-pocket payments and quality of health services) make moving towards merging more difficult. The experience of studied countries showed that one of the source of resistance against and operational challenges of merging HIFs is related to setting new contribution rates for different groups of the insured and modifying the benefit package in a way to satisfy all groups. According to Turkey, to make merging possible, to reduce the resistance of privileged groups, and to increase equity in access to health care services, government should support merging financially and extend the benefit package for underprivileged groups in terms of the range and the depth of health services and even the range of health facilities in which beneficiaries are entitled to get health services they need. As in all countries the size and number of poor people who rely on the government to get health insurance coverage is considerable, this expansion in benefit package can put high financial pressure on the government. Merging should be considered as a policy window to formulate new contribution rates for different group of population bearing in mind the political resistance of privileged groups, improving the financial equity and ensuring financial sustainability of the national single health insurance after merging in the long term. The findings demonstrated that setting new contribution rates for the self-employed and those without coverage can be difficult, redistribution of governmental subsidies from privileged groups enjoying governmental budget in the favor of underprivileged groups and putting more financial obligation on the employees and employers can be challenging.

### Is merging health insurance schemes enough alone?

It is worth mentioning that the merger of HIFs should not be considered as the solution to all health system problems, and we cannot expect a great improvement in the performance of the health system as a result of merger. However, it can be considered as a potential solution in health care financing, and along with other reforms in health sector, it can be much more effective due to the synergy that they create together. Therefore, in Turkey, we see that in order to speed up the effort begun in the 1960s, the health transformation plan (HTP) devised comprehensive strategies to improve the key performances of the health system, such as governance, financing and service provision [11, 33, 37]. The HTP in Turkey had three main initiatives including introduction of the public health insurance scheme, improving public health care and introducing the family physician program, and empowering hospitals to achieve financial and administrative independence [34, 58]. The experience of Turkey, Thailand and South Korea show that if merging as a health financing reform is going to be effective it should be accompanied by other cost-limiting reforms like moving towards close-ended payment methods including capitation, and DRG, emphasizing on PHC services to constrain health care expenditures in the long run. In Turkey, the alignment of demand side and the supply side, made it possible to reach and keep the UHC and increase the access to services, especially for the poor. Evidence shows that although a better insurance coverage improves accessibility, the service package is realized in the presence of the interventions form the supply side [11]. In South Korea as the main payment method is fee-for-service, it is expected that by implementing “Moon Jae-in Care” the total health care expenditures will go up. So it has been advised that Moon Jae-in Care should emphasize more on primary health care and reorganize the health system by moving towards regionalization and reducing the pressure on tertiary health level [25]. Without clinging on restraining health care expenditures reforms it is difficult to keep the advantages which are expected to get from merging.

### Study limitations and strengths

One of the main limitations of this study was the lack of relevant research literature on the merging of health insurance funds in the world and the lack of valid direct and specific reports in this field. In countries where the experience of merging HIFs has been briefly referred to in the literature, including the advantages, disadvantages, and processes used, inadequate relevant credible details and information had been published regarding the topic. Different articles had addressed the experience of merging HIFs partially. This precluded us to cover each aspect of merging HIFs in studied countries in full details. Although we did our best to collect all relevant documents; even we communicated the authors personally via email with the most relevant publications about the topic, still there might have been some documents which we could not find and include in the study.

This study is one of the first studies of its kind to be published in the field of health insurance funds consolidation, and at the same time an attempt has been made to include all valid experiences in countries around the world, so it can be helpful for those who are interested in studying this subject. In addition, the present study has examined and studied in detail the existing experience, which in this regard has provided very valuable information about the different dimensions of the topic in each country. This study tried to open a window for research on the merging of health insurance funds in countries around the world.

## Conclusion

In many countries with health insurance system, there are different schemes for covering different parts of the population which are in part the result of the gradual development of insurance to cover different groups. This fragmentation in health insurance funds usually with different revenues and managerial structures will create differences in the operational procedures and different aspects of insurances. The more and deeper differences between HIFs, the more operational challenges and resistance should be addressed to create a single insurance scheme. This resistance prevented Thailand from achieving the single insurance system they wanted, and despite achieving universal coverage, the harmonization of the three government insurance schemes is still facing challenges and debates.

The merging of HIFs and moving from multiple insurances to a single insurance system is difficult from both politics and implementation viewpoints. As we mentioned, different groups are influenced by reducing the fragmentation, and their interests must be considered while merging the insurances. However, the experience of the these countries showed that political commitment and determination of the government and administrators helped them pursue their goal despite facing oppositions and political challenges, and move toward establishment a single health insurance scheme.

To make the policy of merging HIFs more effective and bring about more positive outcomes for the health sector, it should be accompanied by reforms in the supply side simultaneously.

More documents are required to be published to reveal more details about the experience of merging HIFs in these countries and also other countries especially about the process and different stages of merging health insurance funds.

## Data Availability

The corresponding author will gladly provide any supporting materials upon request.

## Abbreviations

HIFs: health insurance funds
WHO: World Health Organization
OECD: Organization for Economic Co-operation and Development
OOP: Out-of-pocket
NHIC: National Health Insurance Corporation
NHI: National Health Insurance
DRG: Diagnosis Related Group
SIO: Social Insurance Organization
GERF: Government Employees Retirement Fund
SSK: Social Insurance Organization
GHIS: General Health Insurance Scheme
HTP: Health Transformation Plan
UHC: Universal health coverage
MEDULA: Electronic information management system for the Social Security Institution
GDP: Gross domestic product
CSMBS: Civil Servant Medical Benefit Scheme
SSS: Social Security Scheme
VHCS: Voluntary Health Card Scheme
CSMBS: Civil Servant Medical Benefit Scheme
HCP: Health Card Project
NHSO: National Health Security Office
UCS: Universal Coverage Scheme
MOPH: Ministry of Public Health
HMO: Health Maintenance Organization
NHIB: National Health Insurance Bill
NHIS: National Health Insurance System
